# 2224. Aminopenicillins versus Non-aminopenicillins for Treatment of Enterococcal Cystitis

**DOI:** 10.1093/ofid/ofac492.1843

**Published:** 2022-12-15

**Authors:** Jamison Montes de Oca, Michael Veve, Rachel M Kenney

**Affiliations:** Norton Hospital, Louisville, Kentucky; Henry Ford Health, Detroit, Michigan; Henry Ford Hospital, Detroit, Michigan

## Abstract

**Background:**

Aminopenicillins achieve urinary concentrations that overcome aminopenicillin resistance in *Enterococcus* spp. lower urinary tract infections (UTI). With this rationale, our clinical microbiology laboratory discontinued routine identification on Enterococcus urine isolates in 2012 and report “aminopenicillins are predictably reliable for uncomplicated enterococcal UTI”. The study objective was to compare outcomes of patients treated with aminopenicillins (AP) versus non-aminopenicillins (NAP) for enterococcal cystitis.

**Methods:**

IRB approved, retrospective cohort of adults hospitalized with symptomatic enterococcal cystitis from 2013-2021. Exclusion: definitive *E*. *faecalis*, enterococcal UTI in past year, review of systems unavailable/altered mental status, urinary instrumentation except for stent/catheter, systemic infection, fever, genitourinary trauma, renal transplant. Primary endpoint: clinical and microbiologic success at 14 days, defined as resolution of symptoms without new symptoms and no repeat culture growth of index organism. Logistic regression evaluated characteristics associated with 14-day failure. Sample size of 178 calculated for non-inferiority using α=0.025, β=0.8, 15% non-inferiority margin.

**Results:**

178 subjects included, 89 AP, 89 NAP . VRE identified: 73 (82%) AP and 76 (85%) NAP (P=0.50). Amoxicillin (36/89, 40%) and ampicillin (36/89, 40%) used most in AP. Linezolid (41, 46%) and fosfomycin (30, 34%) most common agents in NAP. 14-day clinical composite endpoint was no different between AP and NAP groups, respectively (74 [83%] vs. 73 [82%], P=0.84). No variables independently predicted failure (Table 1). Non-inferiority analysis for AP vs. NAP for 14-day composite: mean difference 1.1% (-0.14 to 0.127, 97.5% CI; P=0.42). Definite E. faecium subgroup: 14-day clinical composite success for was no different between AP and NAP groups, respectively (27/34 [79%] vs. 53/66 [80%], P=0.916).

**Figure:**
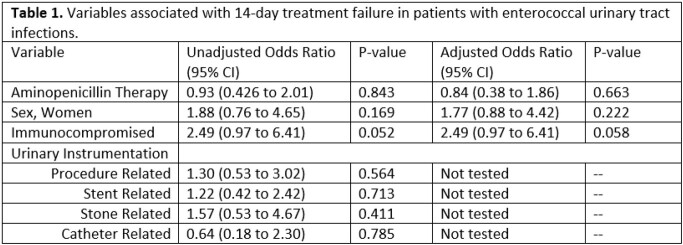

**Conclusion:**

AP were non-inferior to NAP, supporting their use as first-line therapy for cystitis due to enterococci, including *E*. *faecium*. This stewardship strategy has implications to preserve VRE active therapies (e.g. linezolid) for serious infections.

**Disclosures:**

**All Authors**: No reported disclosures.

